# Early-Onset Drug Hypersensitivity Syndrome in a Man With Pneumonia Due to Pre-sensitization to Olanzapine

**DOI:** 10.7759/cureus.26374

**Published:** 2022-06-27

**Authors:** Azusa Sogo, Hiroshi Horiuchi, Takashi Ueda, Hiroshi Miyazaki, Ryosuke Furuya

**Affiliations:** 1 Department of Critical Care and Emergency Medicine, National Hospital Organization Yokohama Medical Center, Yokohama City, JPN; 2 Department of Dermatology, National Hospital Organization Yokohama Medical Center, Yokohama City, JPN

**Keywords:** skin rash, adverse drug reaction, olanzapine, drug-induced hypersensitivity syndrome, drug hypersensitivity syndrome

## Abstract

Drug hypersensitivity syndrome (DHS) generally starts two weeks to two months after administration of certain drugs. Olanzapine has been reported to cause drug reaction with eosinophilia and systemic symptoms (DRESS), but rarely causes drug-induced hypersensitivity syndrome (DIHS). A 49-year-old schizophrenic man was hospitalized for pneumonia and developed DIHS/DRESS 10 days after starting olanzapine. Although reactivation of human herpesvirus 6, which is one of the diagnostic criteria for DIHS, was not confirmed, the diagnostic criteria for DRESS were met. The patient may have developed early-onset DIHS/DRESS because he was sensitized to olanzapine prior to hospitalization.

## Introduction

Drug hypersensitivity syndrome (DHS) is a severe drug eruption that is reported to occur within two weeks to two months after the administration of certain drugs [1]. In Japan, the diagnostic criteria for drug-induced hypersensitivity syndrome (DIHS) are used [2], while in other countries the diagnostic criteria for drug reaction with eosinophilia and systemic symptoms (DRESS) are used [3]. Although olanzapine has been reported to cause DRESS, there have been few reports of it causing DIHS. This report describes the case of a Japanese man who developed early-onset DIHS/DRESS 10 days after starting olanzapine.

## Case presentation

**Figure 1:**
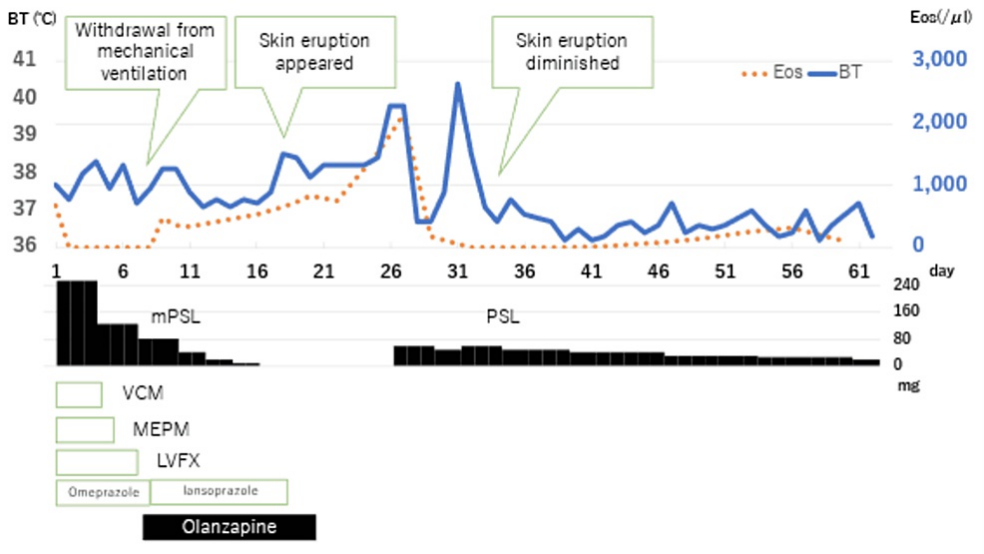
Clinical course of the patient’s disease according to body temperature (BT) and the eosinophil count. The red dotted line shows the BT, and the blue line shows the eosinophil count. BT, body temperature; Eos, eosinophil count; day, days since admission; mPSL, methylprednisolone; PSL, prednisolone; VCM, vancomycin; MEPM, meropenem; LVFX, levofloxacin.

**Figure 2:**
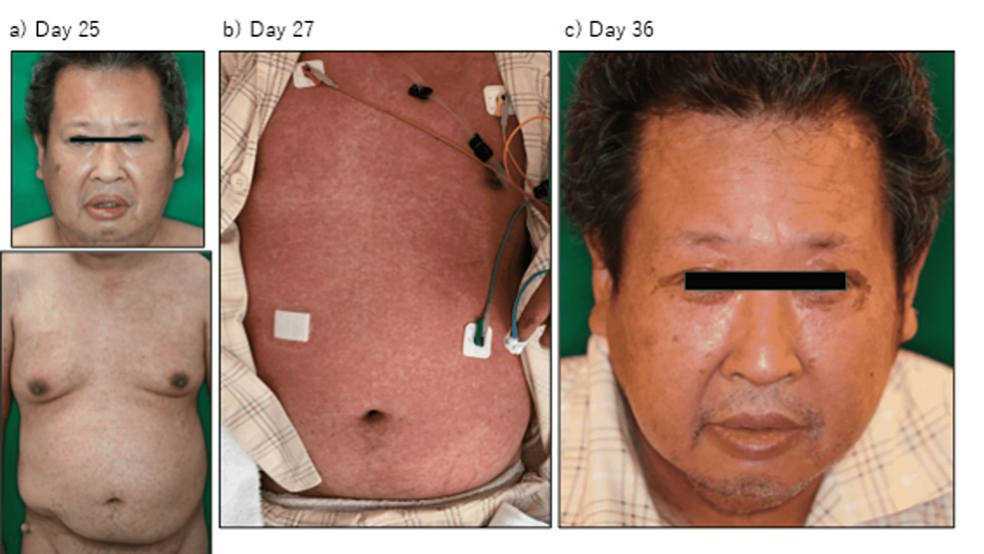
Rash thought to have been caused by olanzapine-induced hypersensitivity. Skin eruption five days (a) and seven days (b), and 16 days (c) after the discontinuation of olanzapine (on days 25, 27, and 36 of hospitalization, respectively). Despite the discontinuation of olanzapine, the skin lesions worsened initially but subsequently resolved following the administration of steroids. Even though skin eruption began to improve on day 34, facial edema and erythema with desquamation of cheeks and periorbital area were still observed on day 36 (c).

**Figure 3:**
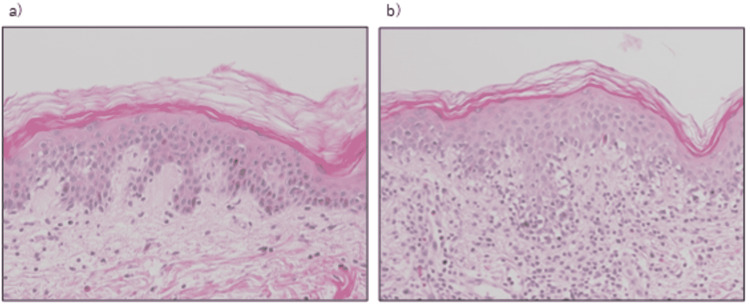
Histopathologic findings of skin with no lesion (a) and skin with erythema (b). Findings of interface dermatitis with basal vacuolization, lymphocyte infiltration, and shedding of melanocytes from the basal layer to the dermis layer were observed (b).

A 49-year-old man with a history of bronchial asthma and schizophrenia was admitted to hospital with a one-month history of dyspnea and a two-day history of fever. He was admitted to the intensive care unit on a ventilator (day 1). On admission, severe acute respiratory syndrome coronavirus 2 (SARS-CoV-2) antigen and polymerase chain reaction (PCR) tests were negative. Urinary pneumococcal antigen and urinary Legionella antigen tests were negative, and the *Mycoplasma pneumoniae* immunoglobulin M (IgM) titer, measured using a passive particle agglutination test, was <40. He was diagnosed with severe bacterial pneumonia, complicated by heart failure, and was treated with meropenem for five days, vancomycin for four days, and levofloxacin for seven days. Methylprednisolone (mPSL) was started at 250 mg/day for bronchial asthma exacerbation and tapered off. Sputum culture on admission showed only normal flora, but atypical pneumonia caused by *Chlamydophila pneumoniae* could not be ruled out because the patient’s *Chlamydophila pneumoniae* immunoglobulin G (IgG) titer was 90 EIU on day 16. He had been started on olanzapine for schizophrenia two months prior to admission. On day 8, when he was withdrawn from the ventilator, oral olanzapine was resumed (Figure 1). On day 9, omeprazole, which was started on day 1 for steroid ulcer prevention, was switched to oral lansoprazole. Eosinophil count elevated to 484/μl on the same day. On day 18, he developed a fever of 38.5℃ and papular erythema appeared on his abdomen and both legs. The cause of the rash was suspected to be a drug eruption, and so lansoprazole and olanzapine were discontinued on days 18 and 20, respectively. After discontinuation of both drugs, the patient continued to have fever in the 38℃ range, liver dysfunction, and eosinophilia, but the rash worsened, and facial involvement was observed (Figure 2). No symptoms of pulmonary involvement were observed. The patient was diagnosed with DIHS/DRESS because he scored 5 on the DIHS diagnostic criteria and 6 on the DRESS diagnostic criteria. Histopathologic findings of interface dermatitis supported the diagnosis (Figure 3). On day 27, mPSL 60 mg/day was started for DIHS/DRESS. On day 29, a human herpes virus 6 (HHV-6) DNA qualitative test was negative and the fever resolved. The mPSL dose was reduced to 50 mg/day on day 31. However, the erythema did not improve, and the mPSL dose was increased to 60 mg/day on day 33 because fever over 38°C reappeared. The rash improved on day 34. Drug lymphocyte stimulation tests (DLST) for lansoprazole and olanzapine on day 22 and day 53 were both negative. Patch tests of olanzapine and lansoprazol were not performed. Following no apparent flare-ups after day 33, steroids were tapered, and the patient was discharged on day 63 on a dose of 20 mg/day of prednisolone.

Steroids were tapered every two to four weeks until the dose had been tapered completely (to 0 mg) on an outpatient basis until day 251. No autoimmune complications were evident on day 251, and the steroids were discontinued. As far as we followed him up, no antipsychotic drugs including olanzapine were reinitiated because his psychiatric symptoms did not worsen.

## Discussion

In this case, the rash and fever are thought to have been due to DIHS/DRESS caused by olanzapine. This case raises two notable points: The onset occurred only 10 days after reinitiating olanzapine, possibly because of sensitization to olanzapine prior to hospitalization. In addition, olanzapine often does not cause reactivation of HHV-6, and as reactivation of HHV-6 is one of the diagnostic criteria for DIHS, this may explain why there have been few reports of DIHS caused by olanzapine in Japan.

DIHS/DRESS can be triggered by upper respiratory tract infection [1], and may have been triggered by the bacterial pneumonia that led to the patient’s hospitalization. Although DLST for lansoprazole and olanzapine on day 22 and day 53 were both negative, lansoprazol had been administered only for nine days when skin eruption appeared, and there were no reports of DIHS/DRESS caused by lansoprazol. Since no other drugs including antimicrobial agents were administered at the time of onset, olanzapine was suspected as the most likely suspect drug. Histopathologic findings supported the diagnosis of DIHS/DRESS. The Pharmaceuticals and Medical Devices Agency in Japan received a total of 23 reports of drug reactions with eosinophilia and systemic symptoms due to lansoprazole from 2007 to 2021, including one death. A causal relationship between lansoprazole and the death could not be confirmed due to insufficient information or the concomitant use of other high-risk drugs for DRESS such as trimethoprim-sulfamethoxazole, vancomycin, and phenobarbital.

DHS usually develops within two weeks to two months after the drug is initiated [1]. A possible reason for the early onset of the disease in this case is the history of olanzapine use prior to hospitalization. Since the number of drugs that can cause DIHS/DRESS is relatively limited, it is important to check the patient's medical history. A diagnosis of DIHS/DRESS should not be ruled out if the onset of symptoms occurs within two weeks of initiating the suspect drug. Recently, three cases of DIHS caused by coronavirus disease 2019 messenger RNA (mRNA) vaccine were reported with the onset two to six days after the initial vaccination [4]. Although there may be differences between mRNA vaccine-induced DIHS and drug-induced DIHS, this suggests that DIHS can develop earlier than is generally reported [4].

The patient was followed up as an outpatient until day 251, and did not experience a relapse after remission or complications such as autoimmune disease that can occur as long-term complications of DIHS/DRESS. The first reported case of DIHS/DRESS caused by olanzapine had a skin rash, fever, elevated eosinophils, and liver damage 60 days after olanzapine initiation, which rapidly improved after olanzapine was discontinued [5]. This case, which required long-term steroid administration even after olanzapine discontinuation, is more typical of DIHS/DRESS, which is reported to take an average of seven weeks for symptoms to improve [6].

There have been no case reports of DIHS caused by olanzapine in Japan as far as we have searched on PubMed and J-STOR. This may be due to the difference between the criteria for DIHS used in Japan and the diagnostic criteria for DRESS, which have been used overseas. In fact, in this case, reactivation of HHV-6 was not proven, so the definitive criteria for DIHS were not met, but the DRESS criteria, European Registry of Severe Cutaneous Adverse Reactions to Drugs (RegiSCAR) score of 6, met the criteria. HHV-6 reactivation has been reported to be detectable in more than 70% of patients with DIHS/DRESS if the test is performed at the appropriate time, usually 14 to 28 days after the onset of symptoms [1]; however, the case that led to the DIHS/DRESS description being added to the olanzapine package insert in Japan on August 4, 2016 was not accompanied by HHV-6 reactivation and was considered to be an atypical case of DIHS [7]. Although there are no reports of differences in HHV-6 reactivation rates between drugs, the lack of reports of olanzapine-induced DIHS in Japan may be partially due to the fact that olanzapine is unlikely to reactivate HHV-6. It has been reported that Japanese criteria for atypical DIHS have decreased diagnostic sensitivity for definite/probable DRESS, and it has also been reported that more than 40% of patients with severe DRESS did not meet diagnostic criteria [8]. There appears to be consensus among experts that many patients with a typical clinical presentation can be diagnosed with DIHS/DRESS without the need for the HHV-6 reactivation criterion to be present [1]. Other herpes viruses such as Epstein-Barr virus, human herpes virus 7, cytomegalovirus (CMV), and varicella-zoster virus have been reported to reactivate during the course of DIHS/DRESS, and although this is not included in the diagnostic criteria, evaluation for reactivation of these viruses may help to confirm the diagnosis [1]. Although the patient was not tested for CMV reactivation in this case, CMV reactivation in particular has also been reported as a poor prognostic factor [9], and as plasma PCR testing for CMV has been covered by health insurance in Japan since 2020, testing for CMV reactivation may be performed more frequently and be included in the diagnostic criteria for DIHS in the future.

## Conclusions

We encountered a case of DIHS/DRESS that developed 10 days after taking olanzapine. It is important to check the medication history of patients with suspected DIHS/DRESS because the onset of the disease may occur within two weeks of taking the suspected drug if the patient has taken the drug previously. In addition, in cases where HHV-6 reactivation is not observed in Japan, it is not necessary to adhere to the DIHS diagnostic criteria, and the DRESS diagnostic criteria should also be used as a reference. Further studies are warranted to determine the timing of onset of olanzapine-induced DIHS/DRESS from the initiation of the medication.
